# RID: Evaluation of the Possible Inhibiting Effect of the Proinflammatory Signaling Induced by TNF-*α* through NF-*κβ* and AP-1 in Two Cell Lines of Breast Cancer

**DOI:** 10.1155/2020/2707635

**Published:** 2020-06-22

**Authors:** F. A. Monsalve, A. Rojas, I. Gonzalez, R. Perez, C. Añasco, J. Romero, P. Araya, L. S. Santos, F. Delgado-Lopez

**Affiliations:** ^1^Department of Basic Biomedical Sciences, Faculty of Health Sciences, University of Talca, Chile; ^2^Laboratories of Biomedical Research, Division of Medicine, Universidad Católica del Maule, Chile; ^3^Laboratory of Asymmetric Synthesis, Institute of Chemistry and Natural Products, University of Talca, Chile

## Abstract

Receptor internalization and degradation (RID), is a transmembrane protein coded within the E3 region expression cassette of adenoviruses. RID downregulates the cell surface expression of epidermal growth factor receptor (EGFR), tumor necrosis factor receptor (TNFR), and apoptosis antigen 1 (FAS), causing a reduction of the effects of their respective ligands. In addition, RID inhibits apoptosis by decreasing the secretion of TNF-related apoptosis-inducing ligand (TRAIL) by normal tissue cells. In this article, we report that RID inhibited chemokine expression in human breast cancer cell line MDA-MB-231 but showed no effect in cell line MCF7. These dissimilar results may be due to the different molecular and functional properties of both cell lines. Therefore, it is necessary to replicate this study in other breast cancer cell models.

## 1. Introduction

Breast cancer is one of the most common cancers among women in the United States. The American Cancer Society estimated that in 2020, 268,600 new cases of invasive breast cancer and about 48,530 cases of carcinoma *in situ* will be diagnosed, and 42,170 are expected to die [1]. In Chile, breast cancer reached a mortality rate of 16.6 per 100,000 women in 2018, which locates it in second place after cervical cancer [2].

Epidemiological and molecular evidence have shown that the process of chronic inflammation affects the progression of cancer [3]. Inflammatory mediators such as nuclear factor kappa *β* (NF-*κβ*), vascular endothelial growth factor (VEGF), proinflammatory chemokines such as IL-8, prostaglandins, p53, nitric oxide (NO), reactive oxygen species (ROS), reactive nitrogen species (RNS), and some specific microRNAs (miRNA) have been shown to be associated with pathogenesis of cancer [4].

For instance, NF-*κβ* a key hub in the immune response has emerged as an important endogenous promoter of hepatocellular, colorectal, and breast cancer [5]. During inflammatory response, NF-*κβ* is activated by pathogen-associated molecular patterns (PAMPs), damage-associated molecular patterns (DAMPs), or cytokines such as TNF-*α* and IL-1*β*. Activation of NF-*κβ* has been shown to promote survival in estrogen receptor- (ER-) negative and ErbB2-positive breast tumors, while selective inhibition of this transcription factor results in apoptosis of breast cancer cell lines [6].

It is known that cancer cells and its interaction with the extracellular matrix and other cell types contribute to modulate an inflammatory milieu at the tumor microenvironment. This occurs through the secretion of cytokines/chemokines and growth and proangiogenic factors. Among them, IL-8 a chemokine with proangiogenic functions, whose expression is controlled by NF-*κβ*, has important autocrine and paracrine effects, increasing proliferation and survival of tumor cells; attracts leukocytes; and induces neovascularization, all processes that precede the invasion and metastasis of tumor cells [7]. An observation that is associated with this phenomenon is that all breast cancers express receptors for IL-8, while only 50% of healthy breast tissue expresses CXR-1 or CXR-2 [8]. On the other hand, a high concentration of IL-8 in serum has been found in patients with breast cancer compared to healthy women. This elevation was also correlated with the clinical status of patients suffering from breast cancer [9].

Adenoviruses express several immunoregulatory genes grouped in the early expression cassette 3 (E3) [10] with various functions during infection. The E3-14.7K protein inhibits apoptosis induced by TNF-*α* [11], and the E3-10.4K/E3-14.5K proteins, also known as receptor internalization and degradation (RID), block apoptosis and inhibit secretion of arachidonic acid induced by TNF-*α* [12], reducing the cell surface expression of Fas, EGFR, TRAIL-R1, TRAIL-R2 [13], and TNFR [14] as well as inhibit lipopolysaccharide signaling (LPS), NF-k*β* activation, JNK activation, and IL-8 and MCP-1 secretion, without altering the expression on the cell surface of the TLR-4 receptor in astrocytoma cell line [15].

The analysis of two breast cancer cell lines, MDA-MB-231 and MCF-7, reveals RID's inhibitory capacity of the TNF-*α*-induced signaling pathway that activates NF-*κβ* and IL-8 secretion to the extracellular medium and by the reduction of neovascular processes in endothelial cells HMEC1 in culture.

## 2. Materials and Methods

### 2.1. Cell Cultures

Human astrocytoma, U373 (ATCC® HTB-17™); human mammary adenocarcinoma, MDA-MB-231 (ATCC® HTB-26™); and MCF-7 (ATCC® HTB-22™) from the American Type Cell Culture Collection (ATCC), Rockville, MD, were grown in 10 mL of Dulbecco's modified Eagle's medium enriched with glucose (DMEM high glucose, Webinar HyClone™) supplemented with GlutaMAX™ plus 10% heat-inactivated bovine serum (SFB) and 1% antibiotic 100x reagent (antibiotic-antimycotic, Gibco® Life Technologies™). Human endothelial-derived cell line, HMEC1 (ATCC® CRL-3243 ™), from the American Type Cell Culture Collection (ATCC), Rockville, MD., were grown in 10 mL of MCDB 131 medium (Gibco® Life Technologies™) supplemented with GlutaMAX™ plus 10% heat-inactivated fetal bovine serum (SFB) and 1% antibiotic 100x reagent (antibiotic-antimycotic, Gibco® Life Technologies™). Incubation of the different cell lines was carried out at 37° C in a humid atmosphere containing 5% CO_2_. The medium was renewed every 72 hrs.

### 2.2. Cells, Viruses, and Stimuli

The mutant adenoviruses AdNull, AdRID, and Ad 14.7K were originally obtained from the laboratory of Dr. William Wold; AdGFP was made at the late Marshall Horwitz laboratory. The adenoviral infection was performed in 12-well plates with a 95% cell confluence in 1 mL of DMEM high-glucose medium, supplemented with FBS and antibiotics. The mutant adenoviruses expressing the vectors are controlled by the CMV (Cytomegalovirus) promoter. Adenoviruses that were used only express the RID protein or only the 14.7K protein, and the null adenovirus used does not express native proteins from the E3 transcription region (Toth et al., 2002). The viruses were added directly to the plate at a concentration of 2,000 particles/cell. The time of adenoviral infection used in this study was 16 to 24 hours. To activate the intracellular signaling pathways, recombinant human TNF-*α* (R & D Systems) was used at a final concentration of 10 ng/mL at different times, as appropriate.

### 2.3. Western Blot

The infected and/or stimulated cells were lysed in 150 *μ*L of electrophoresis loading buffer (50 mM Tris-HCl, 100 mM dithiothreitol, 2% SDS, 0.1% bromophenol blue, and 10% glycerol), boiled at 100°C for 3 minutes, and sonicated. The proteins of the cell lysates were separated in SDS-PAGE gels at a percentage of 10 or 15%, depending on the protein to be tested. Proteins were transferred to 0.2-micron nitrocellulose membranes for 1 hour at room temperature and at a constant current of 150 mA in transfer buffer (20 mM Tris, 150 mM glycine, pH 8.0). After this stage, the membranes were blocked in a solution of 5% skimmed milk in TBS for 30 minutes. Subsequently, the membranes were washed 3 times for 15 minutes each in TBS and incubated in a solution of 1% skim milk in TBS with the primary antibodies (1 : 500 to 1 : 1000 final dilution) anti-I*κ*B*α*, anti-phospho-cjun (purchased from Santa Cruz Biotechnologies), anti-RID*β*, or anti-14.7 (purchased from Genemed Synthesis) overnight at 4°C in constant agitation. The next day, membranes were washed three times for 15 minutes each time in TBS and then incubated for one hour at room temperature with the corresponding secondary antibodies conjugated with HRP (horseradish peroxidase; purchased from Santa Cruz Biotechnologies) at 1 : 3000 dilution factor in a solution of 1% skim milk in TBS. After washing the membranes with TBS to eliminate the excess of antibody, detection was done by chemiluminescence and detected by exposure to X-ray films.

### 2.4. ELISA CXCL8/IL-8

The determination of the cytokine CXCL8/IL-8 was carried out using the commercial kit Quantikine® ELISA from R & D Systems. Both the controls, samples, and standards were quantified in duplicate. Reagents and standards were prepared as described by the manufacturer. 100 *μ*L of the RD1-85 diluent was added to each well of the 96-well format plate. Then, 50 *μ*L of standard and samples (supernatants of diluted cell cultures 1/100) per well were added and subsequently incubated for 2 hours at room temperature. After the incubation period, the total reaction was removed by aspiration and washed 4 times with Wash Buffer (400 *μ*L). 100 *μ*L of conjugate CXCL8/IL-8 reagent was added to all wells and incubated for 30 minutes at room temperature. It was again washed 4 times with Wash Buffer. At the end of this stage, 200 *μ*L of Substrate Solution is added to each well and left at room temperature for 30 minutes protected from light. After this incubation period, 50 *μ*L of Stop Solution per well was added and the optical density was determined at 450 nm.

### 2.5. Angiogenesis HMEC1/ECM-GFR

The Matrigel reagent (BD Biosciences) was used for the angiogenesis assay HMEC1/ECM-GFR (ECM (extracellular matrix); GFR (growth factor reduced)). This was handled according to the manufacturer's instructions, using precooled tubes and tips to prevent premature gelling. The angiogenesis assays were performed in Ibidi *μ*-slides (15 wells per slide). Briefly, the cell culture HMEC1, which had a confluence of 80%, was removed from the culture medium and MCDB131 medium with 2% SFB and antibiotic was added. Cells were incubated for 24 hrs. The next day, 10 *μ*L of Matrigel per well (*μ*-slide plate) was added at a concentration of 8.2 mg/mL. The plate was incubated for 30 to 60 minutes at 37°C to achieve jellification. Then, 50 *μ*L of HMEC1 cells was added at a concentration of 4 × 10^5^ cells/mL and the plate was incubated again at 37°C for 1 hour. After this time, 10 *μ*L of cell supernatants was added under various conditions. The *μ*-slides were incubated for 24 hrs at 37°C in a humid atmosphere containing 5% CO_2_. After the incubation time, *μ*-slides were photographed with the 590CU 5.0M CCD camera coupled to the OLYMPUS CKX41SF microscope.

## 3. Results

### 3.1. Expression of Adenoviral RID Protein in Two Human Breast Cancer Cell Lines, MDA-MB-231 and MCF-7

Breast cancer cell lines, MDA-MB-231 and MCF-7, were used to evaluate RID expression. Human astrocytoma cell line, U373, was used as positive control. RID adenoviral vector only expresses the RID protein. Adenovirus Null does not express proteins encoded in the E3 transcription region. The infection time was between 16 and 24 hrs and m.o.i. was 2000 per cell.

**Figure 1:**
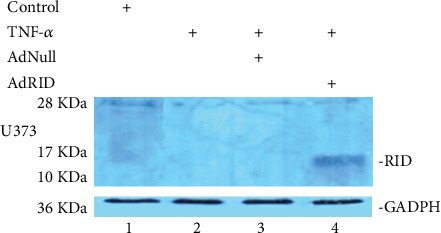
Determination of RID adenoviral protein expression in human astrocytoma U3737 cell line. GADPH: used as loading control.

**Figure 2:**
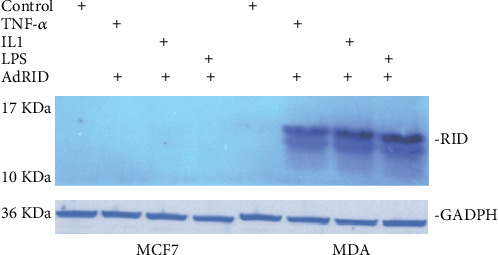
Determination of adenoviral RID protein expression in two human breast cancer cell lines, MCF-7 and MDA-MB-231. The upper western blot shows the expression of the RID protein in the MDA-MB-231 cell line, but not in the MCF7 cell line (data not published by Dr. Fernando Delgado-López). GADPH: used as loading control.

Figure 1 shows the expression of the adenoviral RID protein in the U373 cell line.  Figure 2 shows the expression of RID in MDA-MB-231 but not in MCF7.

### 3.2. Expression of Adenoviral Protein RID: Possible Inhibition of the NF-*κβ* Signaling Pathway in Different Cell Lines Stimulated with TNF-*α*

#### 3.2.1. Activation of NF-*κβ* Signaling Pathway in Cell Lines U373, MDA-MB-231, and MCF-7

**Figure 3:**
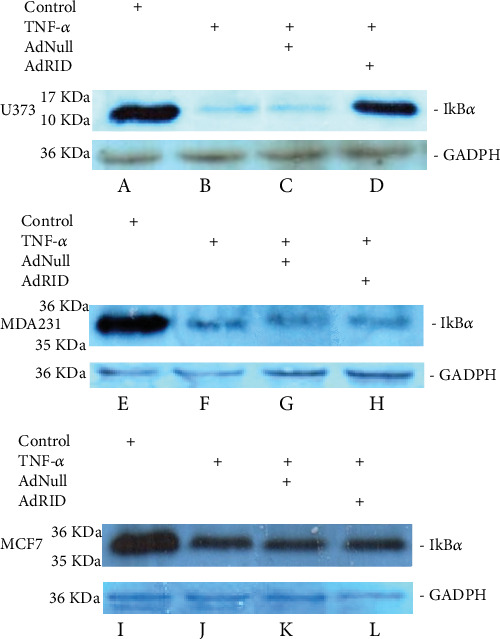
Evaluation of the possible protective effect of the NF-*κβ* pathway of the adenoviral protein RID in the cell lines of human astrocytoma U373 and breast cancer MDA-MB-231 and MCF-7 stimulated with TNF-*α*. Lane A shows the U373 cell line without stimulation. The band of the I*κ*B*α* protein is observed. Lane B shows the effect of TNF-*α* stimulation on the U373 cell line not infected with Adenovirus. Lanes C and D show the U373 cell line infected with Adenovirus Null and RID, respectively. Lane E shows the MDA-MB-231 cell line without stimulation. Lane F shows the effect of stimulation with TNF-*α* in this cell line not infected with Adenovirus. The I*κ*B*α* protein is degraded. Lanes G and H show infection of MDA-MB-231 cells with Adenovirus Null and RID, respectively. Lane I shows the MCF-7 cell line without stimulation. Lane J shows the effect of TNF-*α* stimulation on the MCF-7 cell line not infected with Adenovirus. The I*κ*B*α* protein is degraded. Lanes K and L show the MCF-7 cell line infected with Adenovirus Null and RID, respectively. GADPH: used as loading control.

In the absence of TNF-*α* stimulation, there was a clear band associated with the I*κ*B*α* protein in U373 cells (lane A of Figure 3). However, when U373 cells were stimulated with TNF-*α*, the I*κ*B*α* protein is degraded within 10 minutes, demonstrating NF-*κβ* activation (lane B of Figure 3). Lanes C and D of Figure 3, corresponding to U373 cells stimulated with TNF-*α* and infected with AdNull and RID, respectively, show RID's inhibitory effect. With respect to the activation of the NF-*κβ* pathway in the MDA-MB-231 cell line, lane E of Figure 3 corresponds to the negative control since TNF-*α* was not added to the culture medium. Lane F of this figure shows the effect of TNF-*α* stimulation on the MDA-MB-231 cell line not infected with Adenovirus. It shows that the I*κ*B*α* protein is degraded because of NF-*κβ* activation. Lane G corresponds to the AdNull MDA-MB-231-infected cells stimulated with TNF-*α*, and lane H corresponds to the AdRID MDA-MB-231-infected cells stimulated with TNF-*α*. It seems that RID does not have a large inhibitory effect on the NF-*κβ* signaling pathway. As mentioned above, Figure 2 shows that AdRID infects and expresses RID in MDA-MB-231 cells; thus, we believe that the absence of inhibitory could be due to a low titre presented by these vectors.

Adenoviral RID was also evaluated in the MCF-7 cell line. As seen in lane I of Figure 3, this lane was used as a negative nonstimulated control; hence, I*κ*B*α* is detected. In lane J of this figure, the I*κ*B*α* protein is not degraded after stimulation with TNF-*α* in uninfected cells. Lane K shows stimulation with TNF-*α* in the MCF-7 cell line infected with AdNull. This Adenovirus does not affect the NF-*κβ* signaling pathway, and, finally, in lane L of Figure 3, it is observed that the RID protein does not affect the NF-*κβ* signaling pathway induced by TNF-*α*, since the band associated with the I*κ*B*α* protein, is very similar to that observed in lane J of this figure. These results are correlated with the results obtained by Dr. Delgado-López (Figure 2. unpublished data), so our mutant viruses do not have the ability to infect cells of the MCF-7 line.

### 3.3. Secretion of IL-8 Induced by TNF-*α*

Several groups have shown that cell lines derived from gliomas produce chemokines in response to proinflammatory stimuli [16]. These observations have been extended to other cell types, such as breast cancer cell lines [17]. Earlier results show that RID inhibits the secretion of the chemokines IL-8 and MCP-1 in the U373 cell line [18].

**Figure 4:**
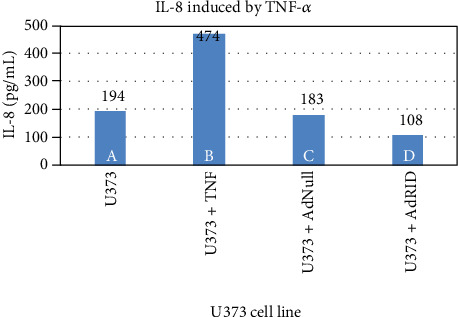
Accumulation of IL-8 induced by TNF-*α* in the supernatant of the human astrocytoma U373 line.

**Figure 5:**
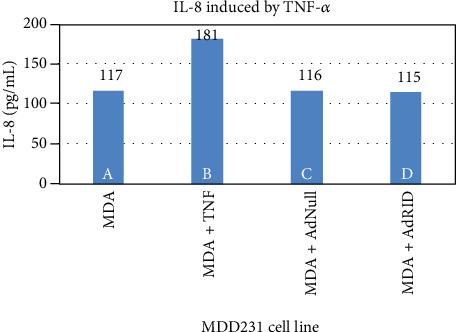
Accumulation of IL-8 induced by TNF-*α* in the supernatant of the human breast cancer line MDA-MB-231.

**Figure 6:**
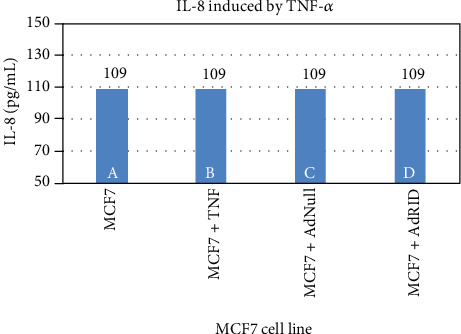
Accumulation of IL-8 induced by TNF-*α* in the supernatant of the human breast cancer line MCF-7.

We then evaluated RID effects on the accumulation of IL-8 in the supernatant of breast cancer cell lines.  Figure 4 reproduces what was previously published in human astrocytoma U373 [15], in which the inhibitory effect of RID on the production of IL-8 induced by TNF-*α* is shown. Column A of Figure 4 shows the accumulation of IL-8 in the supernatant of the U373 cell line not stimulated with TNF-*α*, while B shows IL-8 accumulation upon stimulation with TNF-*α*. Cells infected with AdNull or AdRID, but not stimulated with TNF-*α*, do not increase the concentration of IL-8 in the cell supernatant (Figure 4, columns C and D, respectively). Figures 5 and 6 show similar experiments but using the breast cancer lines MDA-MB-231 and MCF-7, respectively. On the one hand, column A of Figure 5 shows the accumulation of IL-8 in the supernatant of the MDA-MB-231 cell line not stimulated with TNF-*α*. When MDA-MB-231 cells not infected with Ads were stimulated with TNF-*α*, the accumulation of IL-8 in the cell supernatant increased by 50% above the basal accumulation of IL-8. In the MDA-MB-231 cells infected with AdNull, or with AdRID, but not stimulated with TNF-*α*, the accumulation of IL-8 in the cell supernatant is not increased with respect to the basal secretion of IL-8 (columns C and D of Figure 5, respectively). On the other hand, Figure 6 shows marginal effects of stimulation with TNF-*α* and infection with Ads in the MCF-7 cell line.

### 3.4. Expression of the RID Protein Inhibits the Accumulation of IL-8 in the Cell Supernatant

**Figure 7:**
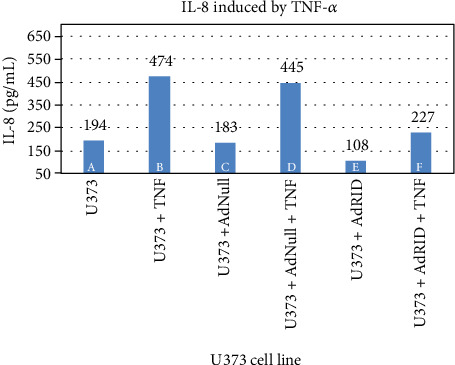
Accumulation of IL-8 induced by TNF-*α* in the supernatant of the human astrocytoma U373 line.

**Figure 8:**
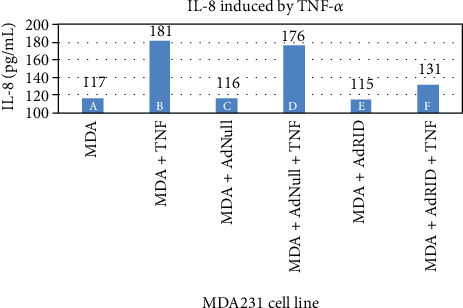
Accumulation of IL-8 induced by TNF-*α* in the supernatant of the human breast cancer line MDA-MB-231.

**Figure 9:**
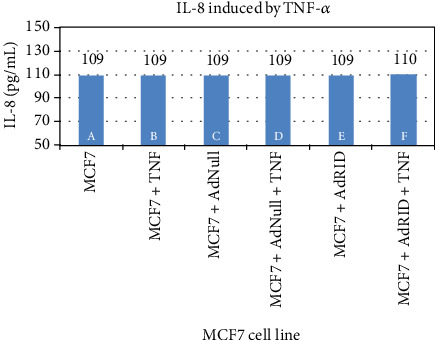
Accumulation of IL-8 induced by TNF-*α* in the supernatant of the human breast cancer line MCF-7.

As seen in Figure 7 column F, U373 cells infected with AdRID and subsequently stimulated with TNF-*α* show a 50% reduction in the concentration of IL-8 in the cell supernatant, in comparison with those cells that were stimulated with TNF-*α* and have no RID expression (Figure 7 column B). Similar experiment was done using the MDA-MB-231 line. As observed in column F of Figure 8, the accumulation of IL-8 in the supernatant of the MDA-MB-231 cell line infected with AdRID and stimulated with TNF-*α* reduced the concentration of IL-8 in approximately 30% compared to those cells only stimulated with TNF-*α* (Figure 8 column B). Finally, as described above, cells of the MCF-7 cell line that were infected with adenoviruses expressing the RID protein did not produce changes in the concentration of accumulated IL-8 in the supernatant. These marginal effects observed in Figure 6 were now analysed under the stimulus of TNF-*α*.  Figure 9 shows the MCF-7 cells exposed to different infection conditions, as well as to stimulation with TNF-*α*. In all the conditions and combinations shown, the concentration of IL-8 accumulated in the supernatant of the MCF-7 cells did not show a modification with respect to the basal condition. All the cell lines U373, MDA-MB-231, and MCF-7 were infected with AdNull, as a negative control for RID expression. As seen in Figures 7, 8, and 9, infection with the Adenovirus Null and subsequent stimulation with TNF-*α* do not reduce the concentration of accumulated IL-8 in the supernatant of the treated cells.

### 3.5. Effect of Conditioned Media on Microvasculature Formation of Human Endothelial Cells HMEC1

The angiogenesis assay was performed on *μ*-slide plates of Ibidi with Matrigel reagent reduced in growth factor with HMEC1 cells.

**Figure 10:**
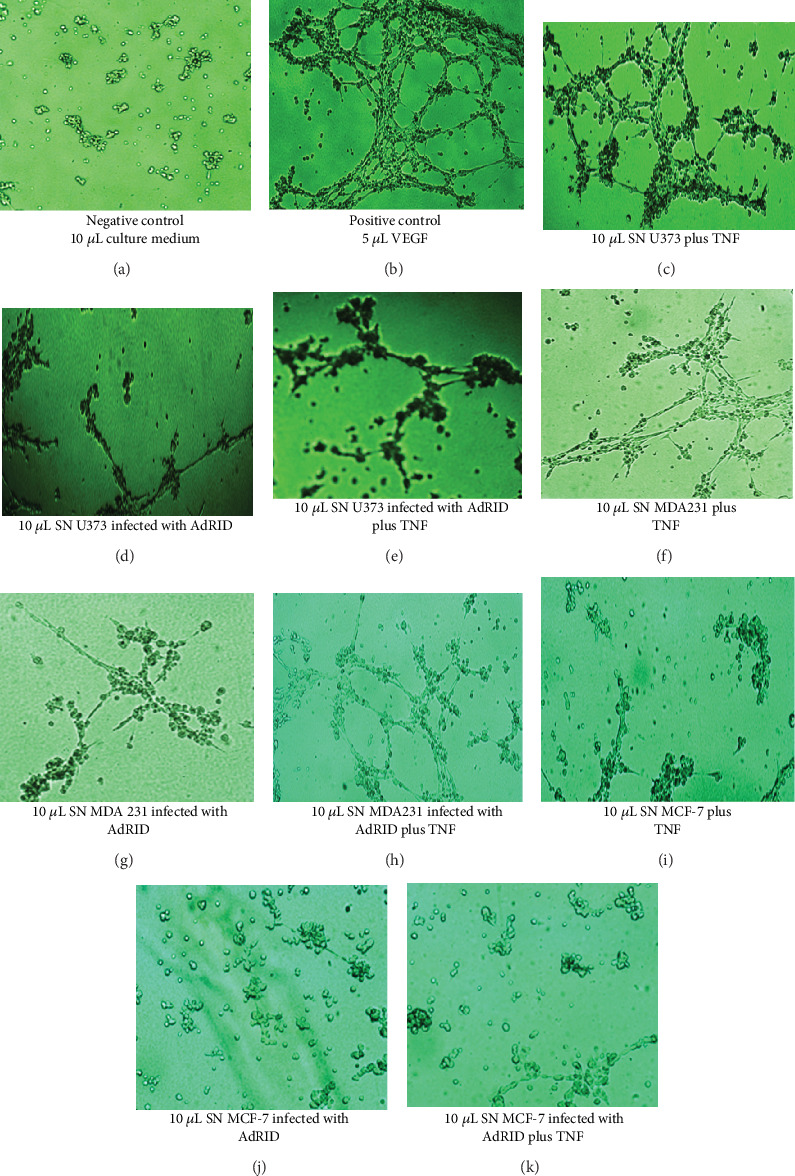
Effect of supernatants of cell lines U373, MDA-MB-231, and MCF-7 stimulated with TNF-*α* on neovascularization in culture of human endothelial cells HMEC1: (a) addition of 10 *μ*L of culture medium MCDB131 supplemented with 10% SFB and 1% Anti-Anti 100x reagent; (b) addition of 5 *μ*L of VEGF reagent; (c) addition of 10 *μ*L of SNs of the U373 cell line stimulated with TNF-*α*; (d) addition of 10 *μ*L of SNs from the U373 cell line infected with RID Adenovirus but not stimulated with TNF-*α*; (e) addition of 10 *μ*L of SNs from the U373 cell line stimulated with TNF-*α* and infected with the RID Adenovirus; (f) addition of 10 *μ*L of SNs of the cell line MDA-MB-231 stimulated with TNF-*α*; (g) addition of 10 *μ*L of SNs of the MDA-MB-231 cell line infected with RID Adenovirus, but not stimulated with TNF-*α*; (h) addition of 10 *μ*L of SNs of the MDA-MB-231 cell line infected with the RID adenovirus and stimulated with TNF-*α*; (i) addition of 10 *μ*L of SNs of the MCF-7 cell line stimulated with TNF-*α*; (j) addition of 10 *μ*L of SNs of the MCF-7 cell line infected with RID Adenovirus, but not stimulated with TNF-*α*; (k) effect of adding 10 *μ*L of SNs of the MCF-7 cell line infected with the RID Adenovirus and stimulated with TNF-*α*.

Figure 10(a) shows the effect of culture medium MCDB131 as a control in the HMEC1 cell line; (b) shows the effect of the addition of VEGF growth factor on HMEC1 cells in culture. The growth factor VEGF caused an increase both in the quality and the amount of neovascular branches observed in the HMEC1 cells in culture. In (c), the effect of the addition of SN of the U373 cell line stimulated with TNF-*α* on HMEC1 in culture can be observed. The addition of this SN produced neovascular ramifications in the HMEC1 cells. This neovascularization could be due to the high concentration of accumulated IL-8 in the SNs of the U373 cells stimulated with TNF-*α*, as shown above.  Figure 10(d) shows the effect of adding SNs of the U373 cell line infected with Adenovirus expressing the RID protein, but not stimulated with TNF-*α*. This reduced the ability to form neovascular ramifications by HMEC1 cells in culture. Similarly, in (e), the effect of adding SNs of the U373 cell line infected with the Adenovirus expressing the RID protein can be observed and, in addition, they were stimulated with TNF-*α* in the well of the HMEC1 cells in culture; this decreased the ability to form neovascular ramifications in HMEC1 cells in culture. Similarly, the SNs of the MDA-MB-231 cell line under various conditions were added to the HMEC1 cells in culture. In Figure 10(f), the effect of the addition of SNs of MDA-MB-231 stimulated with TNF-*α* on HMEC1 in culture was observed. The addition of these SNs induced neovascular ramifications of the HMEC1 cells in culture.  Figure 10(g) shows the effect of adding SNs of the MDA-MB-231 line infected with AdRID but not stimulated with TNF-*α* on the HMEC1 cells in culture. The SN of these cells infected, but not stimulated, showed a diminished effect to induce neovascular ramifications in HMEC1 cells.  Figure 10(h) shows the effect of adding SNs of the MDA-MB-231-expressing RID protein and treated with TNF-*α*, on HMEC1 cells in culture. These SNs show a lower capacity to form neovascular branches in HMEC1 cells than those observed after the addition of the growth factor VEGF. Finally, SNs of MCF-7 cells were evaluated.  Figure 10(i) shows the effect of adding SNs of the MCF-7 line stimulated with TNF-*α*, on the HMEC1 cells in culture; this induced neovascular ramifications in HMEC1 cells growing in Matrigel.  Figure 10(j) of this figure shows the effect of the addition of SNs of the MCF-7 cell line infected with Adenovirus expressing the RID protein, but not stimulated with TNF-*α*, on the HMEC1 cells. The SNs of these cells had a very reduced ability to generate neovascular branches in HMEC1 cells. Finally, the effect of adding SNs of the MCF-7 cell line infected with the Adenovirus expressing the RID protein and stimulated with TNF-*α* in HMEC1 cells is observed in (k).

## 4. Discussion

Earlier data showed that RID inhibited signaling through two inflammation-related receptors, TNFR and TLR-4, in several human cancer cell lines [15]; these observations were validated by measuring inhibition of NF-*κβ* signaling, JNK signaling, and chemokine secretion. As outlined before, breast cancer has been associated with chronic inflammation. Thus, we decided to test whether RID expression could show similar effects in two human breast cancer cell lines, MDA-MB-231 and MCF-7 [19].

We observed that NF-*κβ* activation by TNF-*α* on MDA-MB-231 cells was not inhibited by RID expression (Figure 3 lane H), a rather interesting observation since RID expression was robust. As shown before, RID's ability to inhibit signaling by TNF-*α* that was observed in U373 cells correlates with a reduction in the secretion of IL-8, quantified in the supernatant of these cells. A smaller reduction in the IL-8 accumulation in MDA-MB-231 supernatants was observed. As seen in Figure 4, the accumulation of IL-8 in the supernatant of U373 cells increases 2.4 times with respect to the basal state when U373 cells were stimulated with TNF-*α*. The accumulation of IL-8 in MDA-MB-231 supernatant, after stimulation with TNF-*α*, increases 1.6 times with respect to the basal state (Figure 5). However, IL-8 levels did not change when MCF-7 cells were stimulated with TNF-*α* (Figure 6).

The expression of RID in MDA-MB-231 reduces the accumulation of IL-8 by 30% (Figure 8). There was no effect in MCF-7 cells. Gest and collaborators identified differences in the aggressiveness of these two breast cancer cell lines, MDA-MB-231 and MCF-7. According to these authors, in the cell line MCF-7, the transcription factor NF-*κβ* would be inactivated, so that TNF-*α* does not have the capacity to stimulate this pathway [20]. As seen in Figure 4, infected U373 cells with Ads expressing RID and stimulated with TNF-*α* showed an IL-8 reduction by 50% approximately.

Figure 10 shows the effect of the SNs of the MDA-MB-231 cells on the HMEC1 line. Figure 10(f) shows the neoformative effect of microvasculature after adding SN of MDA-MB-231 cells stimulated with TNF-*α* on HMEC1 cells in culture. In contrast, when SNs of the cell culture MDA-MB-231 that were infected with Ads expressing the RID protein and stimulated with TNF-*α* were added, the process of neovascularization of the HMEC1 cells was markedly reduced (Figure 10(h)). The growth factor VEGF (vascular-endothelial growth factor) was used as a positive control of the neovascularization process. The addition of this growth factor on the HMEC1 cell line induced a potent neovascularization (Figure 10(b)) of these cells. On the other hand, in the case of the SNs of the cells of the MCF-7 line under different conditions, no neovascularizing effects similar to those described for the SNs of the MDA-MB-231 cell line were observed (Figures 10(i)–10(k)). This result was not unexpected since MCF-7 cell line did not show an accumulation of IL-8 in the extracellular medium, upon TNF-*α* treatment. To corroborate this experiment, the SNs of the U373 cell line were used under various conditions (stimulated with TNF-*α*, with expression of the adenoviral RID protein, and stimulated with TNF-*α* plus the expression of the adenoviral protein RID) on the HMEC1 line in culture. The result was as expected, since the addition of the SNs of the U373 cell line that were only stimulated with TNF-*α* on the HMEC1 cells induced a significant neovascularization of this cell line (Figure 10(c)). Furthermore, when the SNs of the cells of the U373 cell line express RID and were treated with TNF, the process of microvasculature formation of the HMEC1 cells in culture was markedly reduced (Figure 10(e)).

Now, it is evident that we cannot ensure that the effects on angiogenesis are due solely to IL-8, since upon TNF-*α* stimulation, there are many other genes activated by the various signaling pathways triggered by it. To assess the IL-8 contribution in this phenotype, we plan to use specific IL-8 gene silencing in similar experiments. The expression of IL-8 has been detected in breast, brain, and colon tumors, as well as in haematological disorders such as leukaemias and lymphomas. In addition, there is a direct correlation between elevated IL-8 serum levels and disease progression, as reported in clinical studies of breast, colon, ovarian, and prostate cancer [21, 22]. Sing et al. demonstrated that metastatic MDA-MB-231 cells produce much more IL-8 than nonmetastatic cells [23]. Studies have revealed that IL-8 secreted from tumors can act in a paracrine fashion to maintain alteration of the tumor microenvironment, as well as act in an autocrine way to facilitate invasion and resistance through oncogenic, angiogenic and prometastatic signaling [7, 24]. These results complement earlier evidence on RID's inhibitory functions of proinflammatory pathways, prompting the design of preclinical studies to evaluate its potential as new adjuvant therapy for the control of breast cancer.

## Data Availability

Data used to support the findings of this study are available from the corresponding author upon request.
